# Supplementary material for: Information Theory for Biological Sequence Classification: A Novel Feature Extraction Technique Based on Tsallis Entropy
Source: Entropy (Basel). 2022 Oct 1;24(10):1398. doi: 10.3390/e24101398 (PMC9601431; doi:10.3390/e24101398)
Supplement: Supplementary file 1 [file entropy-24-01398-s001.zip › entropy-1887207-supplementary.pdf]

Information Theory for Biological Sequence Classification: A Novel Feature Extraction Technique based on Tsallis Entropy

Robson P. Bonidia [1], Anderson P. Avila Santos [1,3], Breno L. S. de Almeida [1], Peter F. Stadler [4], Ulisses N. da Rocha [3], Danilo S. Sanches [2], and André C.P.L.F. de Carvalho [1]

[1] Institute of Mathematics and Computer Sciences, University of São Paulo, São Carlos 13566-590, Brazil
[2] Department of Computer Science, Federal University of Technology - Paraná, UTFPR, Cornélio Procópio 86300-000, Brazil
[3] Department of Environmental Microbiology, Helmholtz Centre for Environmental Research – UFZ GmbH, Leipzig, Saxony, Germany
[4] Department of Computer Science and Interdisciplinary Center of Bioinformatics, University of Leipzig, Leipzig, Saxony, Germany

Supplementary File: S1

Table S1: Overview of Selected Studies

ID	Title	Journal	Year
S1	PROFEAT: a web server for computing structural and physicochemical features of proteins and peptides from amino acid sequence	Nucleic Acids Research	2006
S2	PseAAC: a flexible web server for generating various kinds of protein pseudo amino acid composition	Analytical biochemistry	2008
S3	Update of PROFEAT: a web server for computing structural and physicochemical features of proteins and peptides from amino acid sequence	Nucleic Acids Research	2011
S4	propy: a tool to generate various modes of Chou's PseAAC	Bioinformatics	2013
S5	PseKNC-General: a cross-platform package for generating various modes of pseudo nucleotide compositions	Bioinformatics	2014
S6	PseKNC: A flexible web server for generating pseudo K-tuple nucleotide composition	Analytical Biochemistry	2014
S7	SPiCE: a web-based tool for sequence-based protein classification and exploration	BMC bioinformatics	2014
S8	protr/ProtrWeb: R package and web server for generating various numerical representation schemes of protein sequences	Bioinformatics	2015
S9	ProFET: Feature engineering captures high-level protein functions	Bioinformatics	2015
S10	Pse-in-One: a web server for generating various modes of pseudo components of DNA, RNA, and protein sequences	Nucleic Acids Research	2015
S11	repDNA: a Python package to generate various modes of feature vectors for DNA sequences by incorporating user-defined physicochemical properties and sequence-order effects	Bioinformatics	2015
S12	Rcpi: R/Bioconductor package to generate various descriptors of proteins, compounds and their interactions	Bioinformatics	2015

S13	DNAshapeR: an R/Bioconductor package for DNA shape prediction and feature encoding	Bioinformatics	2015
S14	repRNA: a web server for generating various feature vectors of RNA sequences	Mol Genet Genomics	2016
S15	Pse-in-One 2.0: an improved package of web servers for generating various modes of pseudo components of DNA, RNA, and protein sequences	Natural Science	2017
S16	POSSUM: a bioinformatics toolkit for generating numerical sequence feature descriptors based on PSSM profiles	Bioinformatics	2017
S17	BioSeq-Analysis: a platform for DNA, RNA and protein sequence analysis based on machine learning approaches	Briefings in Bioinformatics	2017
S18	iFeature: a Python package and web server for features extraction and selection from protein and peptide sequences	Bioinformatics	2018
S19	PROSES: A Web Server for Sequence-Based Protein Encoding	Journal of Comput. Biology	2018
S20	PyBioMed: a python library for various molecular representations of chemicals, proteins and DNAs and their interactions	Journal of Cheminformatics	2018
S21	PyFeat: a Python-based effective feature generation tool for DNA, RNA and protein sequences	Bioinformatics	2019
S22	BioSeq-Analysis2.0: an updated platform for analyzing DNA, RNA and protein sequences at sequence level and residue level based on machine learning approaches	Nucleic Acids Research	2019
S23	Seq2Feature: a comprehensive web-based feature extraction tool	Bioinformatics	2019
S24	iLearn: an integrated platform and meta-learner for feature engineering, machine-learning analysis and modeling of DNA, RNA and protein sequence data	Briefings in Bioinformatics	2019
S25	Physicochemical n-Grams Tool: A tool for protein physicochemical descriptor generation via Chou's 5-step rule	Chemical Biology and Drug Design	2020

Supplementary File: S2

Table S2: Descriptor Group

Group	Initials	Application Group
Amino Acid Composition	AAC	Protein
Pseudo-Amino Acid Composition	PseAAC	Protein
Composition, Transition, Distribution	CTD	Protein
Sequence-Order	SO	Protein
Topological Descriptors	TD	Protein
Conjoint Triad	CT	Protein
Proteochemometric Descriptors	PCM	Protein
Profile-based Features	PF	Protein
Nucleic Acid Composition	NAC	DNA, RNA
Pseudo Nucleic Acid Composition	PseNAC	DNA, RNA
Structure Composition	SC	DNA, RNA, Protein
Sequence Similarity	SS	DNA, RNA, Protein
Autocorrelation	AC	DNA, RNA, Protein
Numerical Mapping	NM	DNA, RNA, Protein
K-Nearest Neighbor	KNN	DNA, RNA, Protein
Physicochemical Property	PP	DNA, RNA, Protein

Supplementary File: S3

- **Group:** This column classifies the feature descriptor in each group shown in Supplementary File: S2;
- **Descriptor:** Feature descriptors found in each study and classified in their respective group;
- **Dimension:** Number of features generated by the descriptor (columns). This column is based on the information contained in the revised studies. The "−" symbol means that the dimension may vary according to the chosen parameter, or there is no such information in the studies.
- **Study:** Which study provides the descriptor.

Table S3: Feature Descriptors Found in All Studies

Group	Descriptor	Dimension	Study
NAC	Nucleotide composition	4	S5, S22, S24
	Dinucleotide composition	16	S5, S22, S24
	Trinucleotide composition	64	S5, S22, S24
	Tetranucleotide composition	256	S5
	Pentanucleotide composition	1024	S5
	Hexanucleotide composition	4096	S5
	Basic kmer	4^k	S10, S11, S13, S15, S17, S20, S22, S24
	Reverse complementary kmer	-	S10, S11, S15, S17, S20, S22, S24
	Increment of diversity	$2k$	S11, S15, S17, S22
	Mismatch	-	S15, S17, S22
	Subsequence	-	S15, S17, S22
	GC-content	1	S21
	AT/GT Ratio	1	S21
	Cumulative skew	2	S21
	kGap	-	S21
	Position-specific nucleotide frequency	-	S22, S24
	Nucleotide Content	7	S23
	Conformational properties	18	S23
	Enhanced nucleic acid composition	18	S24
	Composition of k-spaced Nucleic Acid Pairs	-	S22, S24
AC	Normalized Moreau–Broto	240	S5, S15, S17, S22
	Moran	240	S5, S15, S17, S22
	Geary	240	S5, S15, S17, S22
	Dinucleotide-based auto covariance	$N \cdot LAG$	S10, S11, S15, S17, S20, S22, S24
	Dinucleotide-based cross covariance	$N(N-1) \cdot LAG$	S10, S11, S15, S17, S20, S22, S24
	Dinucleotide-based auto-cross covariance	$N^2 \cdot LAG$	S10, S11, S15, S17, S20, S22, S24
	Trinucleotide-based auto covariance	$N \cdot LAG$	S10, S11, S15, S17, S20, S22, S24
	Trinucleotide-based cross covariance	$N(N-1) \cdot LAG$	S10, S11, S15, S17, S20, S22, S24
	Trinucleotide-based auto-cross covariance	$N^2 \cdot LAG$	S10, S11, S15, S17, S20, S22, S24
PseNAC	Type 1 Pseudo k-tuple nucleotide composition	$4^k + \lambda$	S5, S6
	Type 2 Pseudo k-tuple nucleotide composition	$4^k + \lambda \cdot N$	S5, S6
	Pseudo k-tuple nucleotide composition	$4^k + \lambda$	S10, S11, S15, S17, S20, S22, S24

	Pseudo dinucleotide composition	$16 + \lambda$	S10, S11, S15, S17, S20, S22, S24
	General parallel correlation pseudo dinucleotide composition	$16 + \lambda$	S10, S11, S15, S17, S20, S22, S24
	General parallel correlation pseudo trinucleotide composition	$64 + \lambda$	S10, S11, S15, S17, S20, S22, S24
	General series correlation pseudo dinucleotide composition	$16 + \lambda \cdot N$	S10, S11, S15, S17, S20, S22, S24
	General series correlation pseudo trinucleotide composition	$64 + \lambda \cdot N$	S10, S11, S15, S17, S20, S22, S24
SC	DNA shape features	-	S13
NM	Z-curve theory	-	S21, S22
	Nucleotide Chemical Property	-	S22, S24
	Accumulated Nucleotide Frequency	-	S22, S24
	Electron-ion interaction pseudopotential	-	S22, S24
	Pseudo electron-ion interaction pseudopotential	-	S22, S24
	Binary	-	S22, S24
PP	Dinucleotide physicochemical	-	S22, S23
	Trinucleotide physicochemical	-	S22
SS	BLAST-matrix	-	S22
AAC	Amino acid composition	20	S1, S3, S7, S8, S9, S12, S18, S19, S20, S22, S24
	Dipeptide composition	400	S1, S3, S7, S8, S9, S12, S18, S19, S20
	Tripeptide composition	8000	S4, S8, S12, S18, S20, S22, S24
	Terminal end amino acid count	20	S7
	Amino acid pair	400	S19
	Secondary structure composition	3	S7
	Secondary structure - amino acid composition	60	S7
	Solvent accessibility composition	2	S7
	Solvent accessibility - amino acid composition	40	S7
	Codon composition	64	S7
	Protein length	1	S7
	Overlapping K-mers	-	S9
	Information-based statistics	-	S9
	Basic kmer	20^k	S10, S15, S17, S22
	Distance-based residue	-	S15, S17, S22
	Distance pair	-	S15, S17, S22
	Residue-Couple Model	-	S19
	Composition moment vector	-	S19
	Enhanced amino acid composition	-	S18, S24
	Composition of k-spaced amino acid pairs	2400	S18, S22, S24
	Dipeptide deviation from expected mean	400	S18
	Grouped amino acid composition	5	S18, S22, S24
	Enhanced grouped amino acid composition	-	S18, S24
	Composition of k-spaced amino acid group pairs	150	S18, S22, S24
	Grouped dipeptide composition	25	S18
	Grouped tripeptide composition	125	S18, S22, S24
	kGap	-	S21
	Position-specific nucleotide frequency	-	S22

PseAAC	Type 1 PseAAC	$20 + \lambda$	S2, S3, S4, S7, S8, S10, S12, S15, S17, S18, S20, S22, S24
	Type 2 PseAAC	$20 + \lambda \cdot N$	S2, S3, S4, S7, S8, S10, S12, S15, S17, S18, S20, S22, S24
	Dipeptide (or Type 3) PseAAC	420	S2
	General parallel correlation PseAAC	$20 + \lambda$	S10, S15, S17, S22
	General series correlation PseAAC	$20 + \lambda \cdot N$	S10, S15, S17, S22
	Pseudo K-tuple reduced AAC (type1 to type16)	-	S18, S24
AC	Normalized Moreau–Broto	240	S1, S3, S4, S7, S8, S12, S18, S20, S22, S24
	Moran	240	S1, S3, S4, S7, S8, S12, S18, S20, S22, S24
	Geary	240	S1, S3, S4, S7, S8, S12, S18, S20, S22, S24
	Auto covariance	-	S10, S15, S17, S22
	Cross covariance	-	S10, S15, S17, S22
	Auto-cross covariance	-	S10, S15, S17, S22
CTD	Composition	21	S1, S3, S4, S7, S8, S9, S12, S18, S19, S20, S22, S24
	Transition	21	S1, S3, S4, S7, S8, S9, S12, S18, S19, S20, S22, S24
	Distribution	105	S1, S3, S4, S7, S8, S9, S12, S18, S19, S20, S22, S24
SO	Sequence-order-coupling number	60	S1, S3, S4, S8, S12, S18, S20, S22, S24
	Quasi-sequence-order	100	S1, S3, S4, S7, S8, S12, S18, S20, S22, S24
TD	Topological descriptors	405	S3
PF	Signal average	-	S7
	Signal peaks area	-	S7
	PSSM (Position-Specific Scoring Matrix) profile	-	S8, S12, S15, S16, S17, S18, S22, S24
	Profile-based Physicochemical distance	-	S15, S17, S22
	Distance-based Top-n-gram	-	S15, S17, S22
	Top-n-gram	-	S15, S17, S22
	Sequence conservation score	-	S17, S22
	Frequency profiles matrix	-	S22
CT	Conjoint Triad	343	S8, S12, S18, S19, S20, S22, S24
	Conjoint k-spaced triad	$343 \cdot (k + 1)$	S18, S24
PCM	Principal components analysis	175	S8, S12
	Principal components analysis (2D and 3D)	4025	S8
	Factor analysis	175	S8, S12
	Factor analysis (2D and 3D)	4025	S8
	Multidimensional scaling	175	S8, S12
	Multidimensional scaling (2D and 3D)	4025	S8
	BLOSUM and PAM matrix-derived	175	S8, S12, S18, S22, S24
	Biophysical quantitative properties	-	S9

	Amino acid properties	-	S12
	Molecular descriptors	-	S12
SS	Gene Ontology (GO) similarity	-	S12
	Sequence Alignment	-	S12
SC	Secondary structure	-	S17, S18, S22, S24
	Solvent accessible surface area	-	S17, S18, S22, S24
	Secondary structure binary	-	S18, S22, S24
	Disorder	-	S9, S18, S24
	Disorder content	-	S18, S24
	Disorder binary	-	S18, S24
	Torsional angles	-	S18, S24
NM	Binary	-	S18, S22, S24
	Orthonormal encoding	-	S19
	6-dimension One-hot method	-	S22
KNN	K-nearest neighbor for proteins	60	S18, S24
PP	AAindex	-	S9, S18, S22, S24
	Z-scale	-	S18, S22, S24
	Physicochemical n-Grams	-	S25
